# Massive Splenic Infarction: A Rare Entity in a Female Teenager

**DOI:** 10.7759/cureus.95611

**Published:** 2025-10-28

**Authors:** Margarida Moreno Fernandes, Mariana Rodrigues Neto, Andreia Dias Preda, Teresa Pena Fernandes, Joana Rodrigues

**Affiliations:** 1 Pediatrics and Neonatology, Unidade Local de Saúde Gaia - Espinho, Vila Nova de Gaia, PRT

**Keywords:** abdominal pain, desmopressin, epstein-barr virus, infectious mononucleosis, myotonic dystrophy type 1, splenic infarction, steinert’s disease

## Abstract

Splenic infarction is an uncommon, likely underdiagnosed cause of abdominal pain in pediatric patients, with a nonspecific clinical presentation. It is typically associated with hematologic, thromboembolic, or infectious conditions. Epstein-Barr virus (EBV) has rarely been implicated in its pathogenesis. We report a case of massive splenic infarction in a female teenager with Steinert's myotonic dystrophy. This case raises the possibility that desmopressin and Steinert's myotonic dystrophy may have contributed to a prothrombotic state during acute EBV infection.

## Introduction

Splenic infarction is an extremely rare condition in children and adolescents, with an estimated frequency of less than 0.016% among hospital admissions and a true prevalence unknown and likely underestimated due to its nonspecific clinical presentation [1-3]. Splenic infarction is defined as the ischemic necrosis of splenic tissue resulting from occlusion of the splenic artery or its branches. It is usually associated with predisposing factors such as hematologic diseases (particularly sickle cell disease), cardioembolic sources, hypercoagulable states, or infectious etiologies [1].

Among infectious causes, Epstein-Barr virus (EBV) infection has been increasingly recognized as a rare but relevant etiology of splenic infarction. Fewer than 40 pediatric cases have been reported to date, typically occurring within the first two weeks of primary infection and often in otherwise healthy teenagers [4-7]. The proposed mechanisms include transient hypercoagulability, splenic enlargement causing vascular compression, and endothelial injury mediated by viral replication.

Steinert's myotonic dystrophy (myotonic dystrophy type 1) is a multisystemic autosomal dominant neuromuscular disorder characterized by myotonia, muscle weakness, early-onset cataracts, and systemic manifestations. Although thromboembolic events are rare in Steinert's myotonic dystrophy, cases of clinical thromboembolic events such as deep vein thrombosis, pulmonary embolism, and stroke have been described [8-11].

Desmopressin (DDAVP), a synthetic vasopressin analog used for nocturnal enuresis, promotes endothelial release of von Willebrand factor and factor VIII. Although generally safe, it has been associated with isolated thrombotic complications, particularly in predisposed individuals or when combined with other prothrombotic factors [12].

In this report, we present a rare case of massive splenic infarction in a female teenager with Steinert's myotonic dystrophy, occurring shortly after a primary EBV infection. The patient was on chronic desmopressin therapy. The relevance of this case lies in the unusual association between Steinert's myotonic dystrophy and acute EBV infection leading to massive splenic infarction, suggesting that desmopressin therapy may have amplified an underlying prothrombotic tendency.

## Case presentation

A 15-year-old female presented to the pediatric emergency department with a seven-day history of progressively worsening abdominal pain, exacerbated over the last 48 hours, and a single episode of low-grade fever (maximum axillary temperature of 38.8ºC). She denied diarrhea, vomiting, urinary or respiratory symptoms, trauma, or recent surgery. She had a previous diagnosis of Steinert's myotonic dystrophy and primary nocturnal enuresis and was taking desmopressin as daily medication.

On physical examination, she was afebrile, pale, and complaining of generalized abdominal pain, particularly in the right quadrants, associated with rebound tenderness. There was no palpable organomegaly present. The remaining physical examination was unremarkable.

Regarding the laboratory evaluation, the admission results are shown in Table 1. Peripheral blood smear showed atypical lymphocytes.

**Table 1:** Laboratory results on admission

Laboratory Test	Patient Value	Reference Range
Hemoglobin	12.6 g/dL	12–16 g/dL
White blood cells	17,450/µL	4,500–13,500/µL
Lymphocytes	12,550/µL	1,500–6,500/µL
Platelets	451,000/µL	150,000–400,000/µL
Albumin	2.7 g/dL	3.4–4.8 g/dL
Aspartate Aminotransferase (AST)	114 U/L	4-27 U/L
Alanine Aminotransferase (ALT)	157 U/L	4-23 U/L
Lactate Dehydrogenase (LDH)	693 U/L	140–280 U/L
C-reactive protein (CRP)	12.31 mg/dL	0-0.5 mg/dL
Erythrocyte sedimentation rate (ESR)	58 mm/h	0–20 mm/h

**Figure 1:**
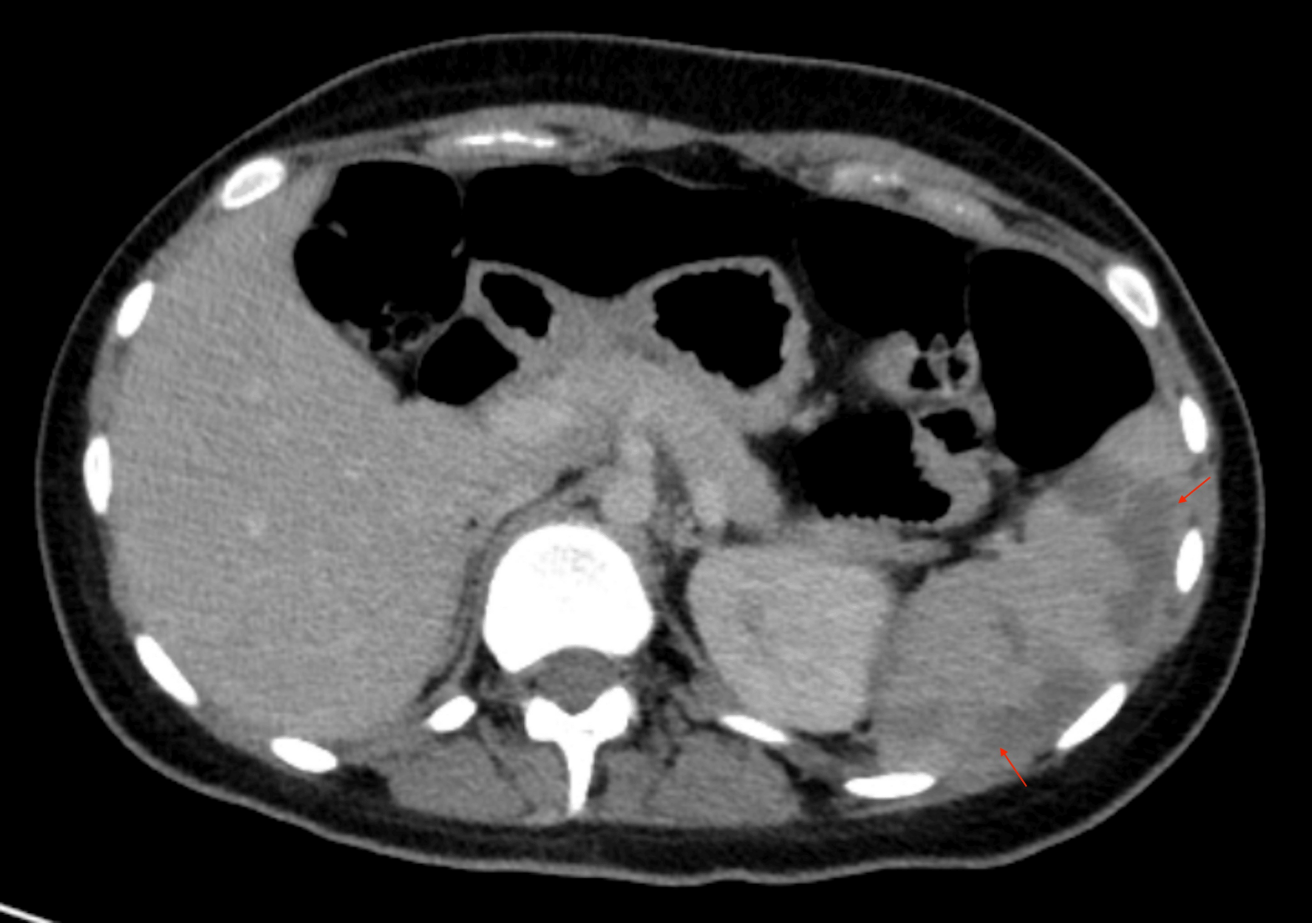
Abdominal CT with extensive splenic infarctions Contrast-enhanced axial CT image of the abdomen demonstrating multiple, wedge-shaped, hypodense areas (arrows) in the spleen.

Abdominal ultrasound, with accuracy limited by intestinal air, revealed homogeneous splenomegaly measuring 13.3 cm and no other findings. An abdominal X-ray suggested possible bowel obstruction, prompting an abdominal computed tomography (CT) scan, which revealed extensive subcapsular wedge-shaped ischemic arterial splenic infarctions involving more than 50% of the parenchyma (Figure 1).

A full microbiological workup is illustrated in Table 2 and showed positive viral capsid antigen (VCA) IgM, negative VCA early antigen (EA) IgG, and negative EBV nuclear antigen (EBNA) IgG, consistent with early acute primary EBV infection.

**Table 2:** Microbiological workup

Laboratory Test	Patient Value	Reference Range
Blood culture	Negative	Negative
Stool bacterial cultures	Negative	Negative
Nasopharyngeal aspirate (PCR panel) - adenovirus, enterovirus, parainfluenza virus types 1–4, human metapneumovirus, influenza A and B, respiratory syncytial virus, and SARS-CoV-2	Negative	Negative
Cytomegalovirus (CMV) IgM and IgG antibodies	Negative	Negative
Anti–Toxoplasma gondii IgM and IgG antibodies negative	Negative	Negative
Herpes simplex virus type I and II (HSV-1 and HSV-2) IgM and IgG antibodies	Negative	Negative
Mycoplasma pneumoniae IgM and IgG antibodies	Negative	Negative
Parvovirus B19 IgM and IgG antibodies	Negative	Negative
Chlamydia pneumoniae IgM and IgG antibodies	Negative	Negative
EBV VCA/EA IgG	0.02 (Negative)	Negative: ≤0.09, Equivocal: 0.09–0.20, Positive: ≥0.20
EBV VCA IgM	3.04 (Positive)	Negative: ≤0.11, Equivocal: 0.11–0.18, Positive: ≥0.18
EBV EBNA IgG	0.00 (Negative)	Negative: ≤0.09, Equivocal: 0.09–0.20, Positive: ≥0.20

The thrombophilia screen, including antiphospholipid antibodies, protein C and S, factor V Leiden, and prothrombin gene mutation, was negative, and the remainder of the coagulation workup is summarized in Table 3. Due to the described association between desmopressin and anticoagulant factors, an extensive thrombotic study revealed elevated levels of von Willebrand factor and factor VIII, most likely induced by the chronic treatment.

**Table 3:** Coagulation workup

Laboratory Test	Patient Value	Reference Range
Activated partial thromboplastin time (aPTT)	32.9 seconds	25.2–35.2 seconds
Prothrombin time (PT)	15.4 seconds	11.5–14.5 seconds
INR (International Normalized Ratio)	1.14	0.9-1.2
D-dimers	4.6 µg/mL	<0.5 µg/mL
Lupus anticoagulant	41.8 seconds	32.4–44.4 seconds
Activated protein C resistance	156.5 seconds	Negative: ≥120 seconds
Protein C activity	84%	70–130%
Protein S activity	55%	55–123%
Antithrombin III (activity)	112%	80–120%
Anti–F-actin antibody	Negative	Negative
Factor VIII activity	309%	50–150%
Von Willebrand factor antigen	151%	50–150%
Prothrombin gene mutation (FII G20210A)	Not detected	Not detected
Factor V Leiden mutation	Not detected	Not detected

Immunophenotyping of peripheral blood revealed increased TCD4 and TCD8 lymphocytes with CD4/CD8 inversion and NK cell lymphocytosis. The electrocardiogram (ECG) was normal, and a transthoracic echocardiogram excluded vegetation or structural heart disease. Due to the extensive infarctions and risk of rupture, she was admitted to the pediatric ward and, after multidisciplinary discussion, was started on treatment with subcutaneous enoxaparin, revealing progressive clinical improvement and no complications, becoming afebrile and pain-free by day four. She repeated the abdominal ultrasound one week later with stable splenic subcapsular infarctions involving more than 50% of the parenchyma.

After 12 days of treatment, anticoagulation was switched to oral apixaban, and the patient was discharged home to outpatient care. She remained asymptomatic, with no abdominal pain or any other symptoms. One month after discharge, a follow-up abdominal ultrasound showed stable splenic lesions, with no new lesions or complications. A follow-up abdominal ultrasound at six months revealed a heterogeneous spleen with hypodense areas suggestive of sequelae of the previous splenic infarctions.

Assuming the hypercoagulability state to be a consequence of the association between desmopressin use and EBV infection, anticoagulation was stopped after six months, and the patient has remained free of symptoms or new events over the last two years.

## Discussion

EBV-related splenic infarction may result from a combination of splenomegaly and transient hypercoagulability, as previously described in the literature based on adult population cases [4,13-16]. Although rare, splenic infarction should be considered in the differential diagnosis of pediatric abdominal pain, especially in the setting of infectious mononucleosis, a common infection in children [4-7].

Although thromboembolic events are uncommon in Steinert's myotonic dystrophy, case reports and small observational studies suggest that endothelial dysfunction, autonomic imbalance, and reduced mobility may contribute to a mild prothrombotic tendency [8-11].

Desmopressin administration appears to be associated with a high risk of thrombotic events, possibly by stimulating the rapid release of endothelial factors, such as an abnormal multimeric form of von Willebrand factor, which may promote platelet aggregation [6]. In this case, the co-administration of desmopressin, with the demonstrated elevation of factor VIII and von Willebrand factor levels, may have exacerbated the prothrombotic state and contributed to the ischemic insult. All three factors (EBV infection, desmopressin, and Steinert's myotonic dystrophy) could have contributed to the pathophysiological mechanism underlying the prothrombotic status. This association, though rare, has not been widely reported in pediatric literature and warrants consideration in similar clinical contexts.

Clinical presentation of splenic infarction is often nonspecific. Abdominal pain, mostly located in the left upper quadrant, is the most common symptom, but pain can be diffuse or referred to the left shoulder, and a high degree of clinical suspicion is necessary, as it can mimic other diagnoses. Abdominal tenderness in our case was more pronounced in the right upper quadrant, probably due to referred pain through shared visceral afferents from splenic irritation. Systemic symptoms, such as fever, chills, nausea, and vomiting, may also be present. Splenomegaly may be present on examination. Nevertheless, 30-50% of patients remain asymptomatic.

Leukocytosis is frequently observed, and it is a suggestive laboratory abnormality. An abdominal CT scan with contrast remains the diagnostic gold standard, revealing wedge-shaped (with the apex pointed toward the hilum and the base of the splenic capsule), peripheral hypodense areas of infarction [2].

Ultrasound may be used initially, but it has lower sensitivity. Possible findings on an abdominal ultrasound include a hypoechoic, wedge-shaped region of splenic tissue with the bright band sign. Evolution of infarction may appear as hyperechoic with retraction of the splenic capsule.

There is currently no consensus regarding the optimal treatment strategy, although most reported cases favor supportive management [1,2,13,16]. Management typically includes supportive care, such as hydration, analgesia, antiemetics, and treatment of the underlying condition.

Pain control should begin with acetaminophen, which is safe and effective for mild to moderate pain, and may be complemented with nonsteroidal anti-inflammatory drugs or opioids when necessary. Early mobilization is encouraged once symptoms improve. In uncomplicated cases, this conservative approach is usually sufficient, as most infarctions resolve spontaneously without sequelae.

Management should also focus on treating the underlying condition to prevent recurrence or extension of the infarction.

Although the role of anticoagulation remains controversial, it may be justified in cases of extensive infarction, documented thrombophilia, prothrombotic triggers, or increased risk of embolic complications. The decision should be individualized according to the etiology and extent of the infarction. In patients with confirmed or suspected thromboembolic or hypercoagulable mechanisms, therapeutic anticoagulation with low-molecular-weight heparin followed by an oral anticoagulant is often recommended. This case also highlights that successful anticoagulation with enoxaparin followed by apixaban may support a more proactive therapeutic approach in selected patients. Massive splenic infarction itself could be regarded as a potential indication for temporary anticoagulation, particularly in the presence of extensive parenchymal involvement, potentially reducing the risk of recurrence. It remains essential, however, to balance the risk of bleeding, particularly splenic rupture, against the potential benefit of preventing infarct progression or embolic phenomena.

After a multidisciplinary discussion, our patient met criteria for treatment with anticoagulation, and the outcome was favorable with full recovery and no complications.

Splenectomy or percutaneous drainage is reserved for complicated cases, such as splenic rupture, abscess formation, or persistent hemorrhage. The prognosis depends on the etiology.

Given the risk of functional hyposplenism, patients should be vaccinated against encapsulated organisms (e.g., *Streptococcus pneumoniae*, *Haemophilus influenzae*, and *Neisseria meningitidis*). Physical activity should be restricted for at least four to six weeks, or until complete radiologic resolution, to prevent splenic rupture or hemorrhage.

## Conclusions

We report a rare case of massive splenic infarction as an unusual complication of primary EBV infection in a teenager with Steinert's myotonic dystrophy on chronic desmopressin therapy. Signs and symptoms are nonspecific, so this case underscores the need for high clinical suspicion in pediatric patients with severe abdominal pain and risk factors for thrombosis. It also underlines the importance of excluding other causes of splenic infarction, including cardiac emboli, hematologic malignancies, and inherited thrombophilias. This case adds to the limited pediatric literature on EBV-related splenic infarction and highlights the importance of considering medication-related prothrombotic factors. We highlight the potential cumulative contributory role of EBV infection, desmopressin, and Steinert's myotonic dystrophy.

While conservative management remains the cornerstone, selected cases, such as those with extensive infarction, may benefit from anticoagulation. Close follow-up is essential to monitor for complications such as splenic rupture or functional asplenia.
